# Current Knowledge Regarding Long-Term Consequences of Pediatric Intensive Care: A Staff Survey in Intensive Care Units in German-Speaking Countries

**DOI:** 10.3389/fped.2022.886626

**Published:** 2022-05-31

**Authors:** Florian Von Borell, Juliane Engel, Felix Neunhoeffer, Florian Hoffmann, Jörg Michel

**Affiliations:** ^1^Department of Pediatric Cardiology and Intensive Care Medicine, Hannover Medical School, Hanover, Germany; ^2^Department of Pediatric Cardiology, Pulmonology and Pediatric Intensive Care Medicine, University Children’s Hospital Tübingen, Tübingen, Germany; ^3^Paediatric Intensive Care and Emergency Medicine, Dr. Von Hauner Children’s Hospital, Ludwig-Maximilians-University, Munich, Germany

**Keywords:** PICS, PICUs, critical care, long-term outcome, sequelae

## Abstract

**Background:**

The Post Intensive Care Syndrome (PICS) describes new impairments of physical, cognitive, social, or mental health after critical illness. In recent years, prevention and therapy concepts have been developed. However, it is unclear whether and to what extent these concepts are known and implemented in hospitals in German-speaking countries.

**Methods:**

We conducted an anonymous online survey in German-speaking pediatric intensive care units on the current state of knowledge about the long-term consequences of intensive care treatment as well as about already established prevention and therapy measures. The request to participate in the survey was sent to the heads of the PICUs of 98 hospitals.

**Results:**

We received 98 responses, 54% of the responses came from nurses, 43% from physicians and 3% from psychologist, all working in intensive care. As a main finding, our survey showed that for only 31% of the respondents PICS has an importance in their daily clinical practice. On average, respondents estimated that about 42% of children receiving intensive care were affected by long-term consequences after intensive care. The existence of a follow-up outpatient clinic was mentioned by 14% of the respondents. Frequent reported barriers to providing follow-up clinics were lack of time and staff. Most frequent mentioned core outcome parameters were normal developmental trajectory (59%) and good quality of life (52%).

**Conclusion:**

Overall, the concept of PICS seems to be underrepresented in German-speaking pediatric intensive care units. It is crucial to expand knowledge on long-term complications after pediatric critical care and to strive for further research through follow-up programs and therewith ultimately improve long-term outcomes.

## Introduction

Over the last decades, there has been a significant reduction in mortality among critically ill patients. At the same time, however, the proportion of patients discharged from intensive care with therapy and disease associated long-term consequences has increased (1). The health consequences of intensive care treatment for adult patients were summarized in 2010 at a conference of the *Society of Critical Care Medicine* in physical, cognitive and mental impairments. The term “*Post Intensive Care Syndrome*” (PICS) was coined to describe this symptom complex resulting from intensive care treatment (2). This also includes frequently reported physical consequences of intensive care treatments such as *critical illness myopathy* and *polyneuropathy*, which occur together in 30–50% of cases (3) and are summarized under the term “*intensive care unit acquired weakness.*” Since long-term consequences after intensive care treatment have also been demonstrated in children (4–7), the clinical picture is gaining attention in the field of pediatric intensive care medicine and is referred to as “*pediatric PICS*” (PICS-p). A concept developed by Manning et al. (8) includes four spheres that are essentially affected and relevant to health: functional impairments, cognitive impairments, losses in emotional experience, and disturbances in social life. In addition to the more frequently discussed functional impairments, pediatric patients also suffer from other long-term consequences such as post-traumatic stress disorder (PTSD), anxiety disorders, developmental deficits, and cognitive impairments (9, 10). The above-mentioned limitations are often accompanied by reductions in health-related quality of life and participation (9, 11, 12). The pathophysiology is multifactorial, due to the different modalities of intensive care treatment and partly unexplained. Particularly in childhood, the individual situation with regard to underlying diseases, but also developmental status and social environment, plays a major role in determining the course of the disease (8, 13). Since intensive care treatment often affects the family environment, which in turn has an influence on the recovery of the patients, research has been turned to affected families in recent years and the term “*PICS family*” (PICS-f) was introduced (14, 15). Due to the critical illness and sometimes long-term care of their child, families can not only reach their economic limits, but also family cohesion as well as the psychological and physical health of individual family members often suffer (16).

It remains unknown whether and to what extent PICS-p and PICS-f are known and implemented in hospitals in German-speaking countries, probably being representative for Central European countries. With a survey of pediatric intensive care units (PICUs) in German-speaking countries, we assessed the current state of knowledge about long-term consequences of intensive care treatment. The data collected will subsequently be used to expand the general body of knowledge and assess the need for further research. Our goal is to raise awareness of pediatric PICS, display its’ underrepresentation, and ultimately push the development of follow-up programs.

## Materials and Methods

For data collection, we conducted an anonymous online survey in German-speaking PICUs (Germany, Austria, Swiss). For this purpose, a catalog of 27 questions was designed with the help of the survey platform LimeSurvey.^1^ the questionnaire was drafted after a thorough review of the current literature. The questionnaire was reviewed by independent pediatric intensive care physicians for clarity of questions, appropriateness of responses, and ease of participation. The questionnaire contained demographic, nominal, cardinal, and open-ended questions. The translated version of the questionnaire is available as Supplementary Material. In addition to the characteristics of the respective intensive care units and the professional status of the respondents, individual levels of knowledge about the clinical picture, perceptions of the current situation on the units as well as obstacles regarding prevention and therapy of PICS-p and PICS-f were assessed. The weighting of individual risk factors and long-term consequences from the respondents’ point of view was surveyed in order to obtain an idea of the current situation in the respective PICUs. Respondents were asked to select risk factors and outcome measures from a list and add others as appropriate. At the beginning of the questionnaire, a short definition of terms (PICS-p; PICS-f) was given.

The request to participate in the survey was sent by e-mail to the heads of the PICUs of 98 hospitals (physicians) in June of 2021: 87 hospitals in Germany, 4 hospitals in Austria, and 7 hospitals in Switzerland. Contacts were obtained through the German Interdisciplinary Association for Intensive Care and Emergency Medicine (DIVI). The contact list was completed by internet research on additional hospitals providing PICUs. The authors assume that the 98 PICUs contacted represent the central European PICU landscape. The heads of the intensive care units could forward the survey-link also to physicians, nursing colleagues, and psychotherapists working at the PICU. A reminder to participate was sent after 6 weeks. All responses received by October 2021 were considered. To ensure the anonymity of the survey, it was not possible to allocate the answers to the respective clinics. Only fully completed questionnaires were included in the analysis and evaluated descriptively. The survey identified the subgroups PICS-experienced and PICS-inexperienced. In order to examine these subgroups with regard to their categorial answers concerning risk factors and outcomes, the statistical calculation was carried out using the Chi-square test. PICS-inexperienced respondents were not excluded from questions on presumed risk factors or long-term outcomes. All statistical analysis were conducted using R statistical computing, version 4.0.3, 2020-10-10 for Mac Os X (Copyright (C) 2020 The R Foundation for Statistical Computing, Vienna, Austria). The study protocol and survey was approved by the Institutional Research Ethics Committee of the Technical University Dresden, Germany. Reporting of the survey was done according to the consensus guidelines for reporting survey studies (CROSS) (17).

## Results

Of 142 responses, 44 were excluded due to incompleteness. A total of 98 questionnaires were included in the analysis. An analysis of unit characteristics revealed that participating respondents came from at least 46 different units. The characteristics of the respondents can be found in Table 1.

**TABLE 1:** Responder characteristics (*N* = 98).

	*n*	%
**PICU type**		
Pure pediatric	69	29.6
Mixed neonatal- pediatric PICU	29	70.4
**Work experience**		
1–5 years	16	16.3
5–10 years	21	21.4
>10 years	61	62.2
**Hospital type**		
University	72	73.5
Other tertiary-care hospital	16	16.3
None of both	10	10.2
**PICS experienced**		
Yes	30	31
No	68	69

Twenty five percent of the respondents stated that they not yet had any contact with the concept of PICS, 31% stated that PICS had a significance in their daily clinical practice. We did not exclude respondents who stated to have no experience with the concept of PICS from further questions as we believe that most clinicians are aware of the potential consequences of intensive care. The question referred to their perceptions, and we wanted to get a picture of the respondents’ suspected long-term problems.

**FIGURE 1:**
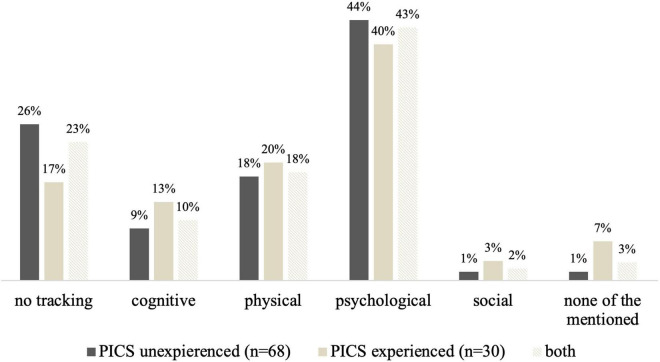
Estimated weighting of long-term consequences after intensive care treatment; *n* = 98.

**FIGURE 2:**
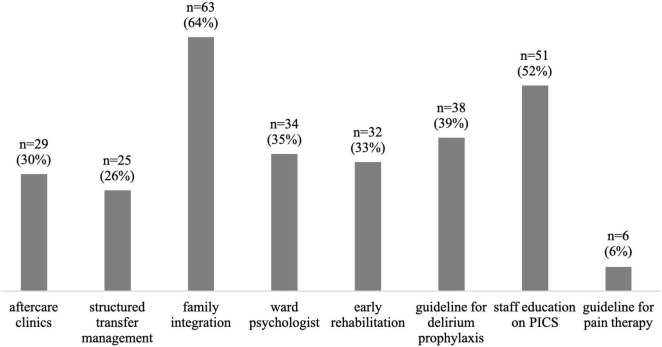
Suitable measures (in terms of effort/benefit) to prevent PICS; *n* = 98 (multiple choice).

On average, respondents estimated that about 42% of children receiving intensive care were affected by PICS-p and 45% of families by PICS-f. Among the respondents’ perceptions PTSD (56%), sleep disturbances (48%), feeding problems (42%), cognitive impairment (34%), and muscular weakness (20%) were the most common long-term consequences of intensive care treatment. 31% of the respondents stated that in the absence of follow-up, it was difficult to determine long-term consequences. A large proportion of respondents (43%) perceived most long-term consequences on a psychological level (Figure 1). Figure 2 presents the most important measures to prevent PICS from the respondents’ point of view.

The most important risk factors were found to be length of stay (56%), delirium and disorientation (53%), number of invasive procedures (28%), lack of family involvement (27%), and severity of illness (24%). The length of stay (55%) and lack of involvement in the child’s care (37%) were also most frequently named as risk factors for family PICS. In addition, the tension between the remaining family at home and the child in the ICU (33%) and an insufficient transfer of information to the family (32%) were frequently identified as risk factors. There was no significant (*p* < 0.05) difference between the perceived risk factors and long-term outcomes stated by PICS-experienced and PICS-inexperienced respondents.

Fifty one percent of the respondents stated that a social history was taken on admission to the ward, 32% stated that the physical condition before admission was assessed in a standardized way. Forty percent of the respondents stated that their PICU did not collect information on social as well as physical, mental, and cognitive conditions prior to admission. Three percent of respondents reported regular and 5% irregular PICS assessments at their unit. Forty two percent of the respondents stated that a standardized physical status assessment was carried out before discharge, 40% stated that the need for social support was assessed. Regular assessment of the need for further psychological support was mentioned by 36%.

Seventy percent of the respondents stated that their ward had a guideline on pain therapy, 66% had a sedation guideline, 56% had a nutrition guideline, and 42% had a guideline on delirium prophylaxis. Eight percent stated to have a guideline on family-oriented treatment and 5% stated to have an implemented guideline for increasing patient comfort. Lack of staff (66%), lack of time (64%), and lack of routine (40%) were named as the most important barriers to the regular implementation of early mobilization, 20% of the responders stated to have a guideline for early mobilization in place.

The existence of a follow-up outpatient clinic was mentioned by 14% of the respondents. A proportion of 54% of respondents said they had no follow-up program at all in their clinic. The most frequent obstacles to the implementation and regular supervision of follow-up programs were a lack of personnel (54%), a lack of awareness of its necessity (46%), and the unclear allocation of tasks between the outpatient and inpatient sectors (41%).

**FIGURE 3:**
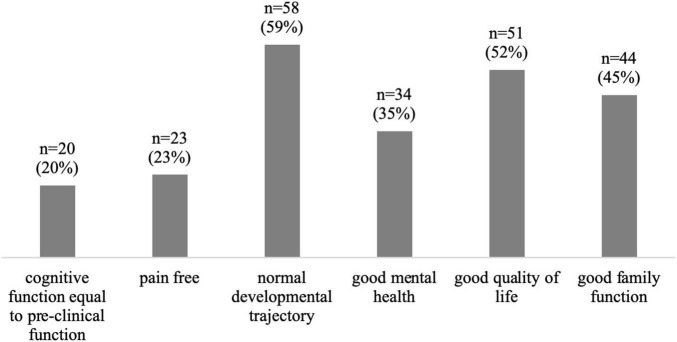
Most frequent selected significant outcome parameters after PICU treatment; *n* = 98 (multiple choice).

Normal age-appropriate development (59%), high quality of life (52%), normal family function (45%), and mental health (35%) were named as the most important parameters for measuring a therapeutic success after discharge (Figure 3).

## Discussion

The aim of this study was to assess the clinicians’ awareness and knowledge on long-term consequences of pediatric intensive care therapy in childhood. A quarter of the respondents had no previous contact with the term “*Post Intensive Care Syndrome*,” only 31% stated that PICS played a role in their daily routine. According to almost half of the respondents, the biggest barrier to the implementation of post-intensive care programs was the lack of awareness of their necessity. The occurrence of PICS-p, on the other hand, was estimated at a mean of 42%. This suggests that there is a discrepancy between the occurrence of long-term impairments and their perception and treatment. What has been shown for adults (2) has also been observed in children after critical care; 6 months after discharge 72% suffer from sleep disorders and 38% from chronic fatigue. In 75% of pediatric patients negative consequences for the health-related quality of life are observed; the PTSD rate is given at about 30% (4, 12, 18–20). About one third of the respondents stated that they lacked knowledge from follow-up to be able to make statements about long-term consequences. However, it is precisely the follow-up and research of late effects that seems to be necessary in order to develop therapy concepts and to avert negative courses (21). Knowledge about risk groups makes targeted prevention and therapy possible, not least in order to be able to use the already scarce resources sensibly (22, 23). Lack of personnel and time were named by the interviewees as the most significant hurdles for PICS prevention and therapy.

In order to assess the individual long-term course, it is not only necessary to provide follow-up care, but also to record the initial condition before intensive care treatment. Many patients already have an impairing underlying disease before their intensive care stay (7, 21, 24). About one third of pediatric patients admitted to a PICU have at least one adverse social determinant (25). Notably, poorer socioeconomic status is correlating with poorer cognitive outcome (26). In our survey, 42% of respondents reported that there was no standardized collection of baseline social, psychological or physical status at their PICU.

Only 14% percent of the respondents reported a follow-up program, this seems low but goes in line with other observations. Williams et al. found in an US focused survey on PICU follow-up programs that 35% of the responding PICUs had a program in place of which only about one quarter was broadly inclusive to a wide range of PICU patients (27). What follow-up after pediatric intensive care should look like in our health system remains unclear. Does the responsibility fall within the scope of professionals within intensive care medicine, who are familiar with the acute illness and therapy and have already gotten to know the patient in their new health condition? Or should an existing outpatient system (pediatrician, outpatient rehabilitation, psychiatrist) deal with it (28)? A feasible option in our health care system could be risk assessment and therapy planning by the staff of intensive care units to enable targeted multidisciplinary outpatient treatment, controlled by pediatricians in ambulatory care (29, 30). To our knowledge, such a system does not yet exist. From our point of view, the development and evaluation of such programs would be important to possibly improve PICS management. To make this possible, patients at-risk must be reliably identified and outcome parameters should be defined. In a Delphi study published in 2020, the following core outcome parameters after critical care were agreed upon: cognitive function, emotional function, communication, general health, painlessness, physical function, survival, and health-related quality of life (31). It should be emphasized that among the respondents of our study, the most frequently selected outcomes tend to be long-term outcomes and that general spheres such as age-appropriate development and good quality of life play an important role. This is in line with previous surveys. In a survey of 85 parents of children receiving intensive care treatment, the respondents indicated important long-term outcomes such as normal appearance and behavior as well as long-term health and lack of developmental problems in addition to short-term outcomes (32). In a survey by Merritt et al. parents and healthcare professionals were both asked about important outcomes. Again, quality of life as well as good function after leaving the hospital were most frequently mentioned by both groups (33). This definition of success of intensive care treatment beyond survival cannot be measured in the short term and in our opinion highlights the need of research in follow-up programs.

A limitation to this study was the impossibility to trace individual survey respondents. Therefore, it is assumable that some respondents work in the same hospital. Thus, we can neither provide a response rate nor can we display the data covering the entire clinical landscape, data on clinical properties can only be considered a tendency. We performed an analysis of the characteristics of the respondents and found that respondents from at least 46 different units participated in the survey. This equals a response rate of at least 47%. We cannot conclude whether this is a representative sample for the German-speaking region. It is possible that there was an over-sampling of PICUs with PICS experience, which would shed an even worse light on the level of knowledge.

Also, the answers reflect the perception of the respondents and not necessarily the practice in the respective PICUs. Because there was a lack of experience with the symptom complex of PICS-p among the respondents, we were not able to provide a sound overview of possible prevention or treatment options. Moreover, the survey has not been validated to assess for PICS management. A next goal with the emergence of new follow-up programs would be to re-survey with a validated questionnaire focusing on risk factors and outcomes as well as program feasibility and barriers. Inherent in the study design is the possibility of the occurrence of response bias. A limited generalizability of our data may be caused by the possibly more frequent survey participation of respondents from hospitals that have already dealt with PICS or have an interest in the topic.

## Conclusion

The survey outlines a picture of current knowledge regarding *Pediatric Post Intensive Care Syndrome* in pediatric intensive care units. Overall, the concept of PICS-p and PICS-f seems to be underrepresented in German-speaking pediatric intensive care units. In contrast, long-term sequelae were observed in an average of more than 40% of the survivors. It is crucial to expand knowledge on long-term complications after pediatric critical care and to strive for further research to develop screening tools and treatment options and therewith ultimately improve long-term outcomes.

## Data Availability Statement

The raw data supporting the conclusions of this article will be made available by the authors, without undue reservation.

## Ethics Statement

The studies involving human participants were reviewed and approved by Ethikkommision der TU—Dresden Fetschstraβe 74 01307 Dresden NR: BO-EK 221042021. Consent was implied by completing the survey.

## Author Contributions

JE, FN, JM, and FV contributed to the study conception and design and wrote the manuscript. FH created the PICU contact list. FV performed material preparation, data collection, and analysis. All authors read and approved the final manuscript.

## Conflict of Interest

The authors declare that the research was conducted in the absence of any commercial or financial relationships that could be construed as a potential conflict of interest.

## Publisher’s Note

All claims expressed in this article are solely those of the authors and do not necessarily represent those of their affiliated organizations, or those of the publisher, the editors and the reviewers. Any product that may be evaluated in this article, or claim that may be made by its manufacturer, is not guaranteed or endorsed by the publisher.

## Footnotes

1
https://www.limesurvey.org/
