# Supplementary material for: Current Knowledge Regarding Long-Term Consequences of Pediatric Intensive Care: A Staff Survey in Intensive Care Units in German-Speaking Countries
Source: Front Pediatr. 2022 May 31;10:886626. doi: 10.3389/fped.2022.886626 (PMC9197504; doi:10.3389/fped.2022.886626)
Supplement: Supplementary file 1 [file Table_1.DOCX]

**PICS Survey**

1. **General information**

A1) The intensive care unit at which you work is a:

- mixed neonatal-pediatric intensive care unit

- pediatric intensive care unit

A2) Which profession do you belong to?

- Nursing without further training in intensive care medicine

- Nursing with further training in intensive care medicine

- Physician and resident in pediatrics

- Specialist in pediatrics without additional qualification in pediatric intensive care medicine

- Specialist in pediatrics with additional qualification in pediatric intensive care medicine

- Psychiatrist

- Psychologist

- Physiotherapist

A3) How much professional experience do you have?

- < 1 year

- 1-5 years

- > 5 years

- > 10 years

A4) How many beds capable of mechanical ventilaton (patients >28 days of life on admission) does the PICU where you work at have?

A5) The hospital you work at is a:

- University hospital

- Tertiary care hospital

- none of the two

A6) Level of neonatal care:

- level I

- level II

- level III

- purely pediatric intensive care unit

1. **Post Intensive Care Syndrome – Background.**

B1) Have you had any contact with the term Post Intensive Care Syndrome?

- yes, it is of importance in my daily work

- yes, I have heard about it, but I am confronted with it only irregularly

- no

B2) Estimate how many patients are affected on average after a PICU stay.

- 0% - 100%

B3) Estimate how many families on average are affected by PICS-f after a PICU stay of their child.

- 0% - 100%

B4) From your point of view/experience, what are the most frequent negative (long-term) consequences of an intensive care treatment?

- chronic fatigue

- sleep disturbances

- depressions

- post-traumatic stress disorders

- muscular weakness

- cardiorespiratory long-term effects

- nutritional problems

- cognitive impairment

- loss of social contacts

- difficult to determine due to lack of follow-up

- I don't know

- other:

B5) At what level do you experience the most limitations in your patients after intensive care therapy?

- on a physical level

- on psychological/emotional level

- on a social level

- on a cognitive level

- on none of the above

- none, because we don’t follow-up

1. **Post Intensive Care Syndrome - Risks.**

C1 In your opinion, what might be the most important risk factors for the occurrence of pediatric post intensive care syndrome (PICS-p)?

- length of PICU stay

- number of invasive procedures

- age

- inadequate / non-patient-friendly information transfer

- delirium / disorientation

- lack of family involvement

- immobilization

- sedatives

- noise / light polution

- severity of illness

- lack of communication

- sepsis

- previous neurological disease

- stressed parents

- lack of aftercare

- lack of psychological support

- don’t know

- other:

C2) In your opinion, what might be the most relevant risk factors for the occurrence of family post intensive care syndrome (PICS-f)?

- length of PICU stay

- number of invasive procedures

- inadequate information sharing

- lack of involvement in the care of the child

- feeling of helplessness concerning the further course of the disease

- severity of illness

- acuity of admission

- unclear outcome

- changed parental roles

- tension between family at home and sick child in the PICU

- new challenges after discharge

- lack of aftercare

- lack of psychological support

- socio-economic status

- I don’t know

- other:

C3) At patient admission in the intensive care unit in which you work you survey for:

- physical condition before admission

- psychological / emotional condition before admission to the unit

- cognitive condition before admission

- social history

- score of current disease severity (e.g. PRISM, PIM, PELODS etc.)

- none of the above

- other:

C4) Does the PICU where you work at currently perform risk assessments in form of a PICS screening?

- yes, regularly

- yes, irregularly

- no

- I don’t know

1. **Post Intensive Care Syndrome – Prevention.**

D1) In your opinion, what could be particularly appropriate (in terms of effort / benefit) measures to prevent PICS-p / PICS-f?

- implementation of post-intensive care aftercare clinics

- implementation of structured discharge or transfer management

- family integration in the intensive care unit

- employment of ward psychologists

- development / implementation of early rehabilitation management

- internal guideline on delirium prophylaxis

- education of medical staff on PICS-p/ PICS-f

- guideline for pain therapy

- other:

D2) The intensive care unit you work at provides:

- a (internal) guideline for early mobilization

- psychological support

- music / art therapy

- family support / social workers

- physiotherapy as a fixed component of patient care integrated into the team

- a guideline for PICS prevention / therapy

- none of the above

- I don’t know

- other:

D3) Are there components of an early rehabilitation program (in terms of e.g. ABCDEF Bundle/ PICUup!/ Liber8...) in the PICU you work at?

- yes, a guideline for delirium prophylaxis

- yes, a guideline for pain therapy

- yes, a guideline on nutrition

- yes, a guideline on sedation / sedation weaning

- yes, a guideline on ventilation and / or ventilation weaning

- yes, a guideline on family-oriented treatment

- yes, a guideline on increasing patient comfort

- no

- I don’t know

- other:

D4) In your opinion, what are the most important transfer issues that should be already addressed at the PICU?

- medication plan

- follow-up treatment

- physiotherapeutic follow-up treatment

- outpatient pain management

- psychological aftercare

- social aftercare

- other:

D5) The PICU where you work has the following informational materials regarding PICS:

- brochures / other media

- informal information provision by staff

- patient education

- none

- I don’t know

- other:

D6) In the intensive care unit where you work, a standardized survey is conducted prior to discharge of:

- physical status / possible functional limitations

- cognitive limitations

- need for social support

- need for psychological support

- none of the above

- I don’t know

- other:

D7) In your opinion, what are the (up to 3) most important barriers to the implementation / regular execution of early mobilization in the PICU where you work?

- time

- lack of staff

- equipment

- lack of necessity

- lack of know-how

- missing routine / protocols

- lack of professions’ accountability

- concerns about patient safety

- sedation

- pain

- lack of patient compliance

- don't know

- other:

1. **Post Intensive Care Syndrome - Therapy.**

E1) Does the PICU where you work at have regular interprofessional rounds involving physical therapists, psychotherapists, parents, etc., with a focus on long-term therapy planning?

- yes

- no

E2) From your point of view, what would be the most important parameters (apart from survival) for measuring a therapeutic success of your PICU patients.

- time to recovery of organ functions

- length of PICU stay

- length of hospital stay

- cognitive function comparable to the pre-hospital situation

- freedom from pain

- normal age-appropriate developmental trajectory after discharge

- good mental health after discharge

- participation in school and social contacts

- high quality of life after discharge

- normal family function, low family stress

- other:

E3) Is there any psychological support in the PICU where you work at?

- yes, for the patients, a ward psychologist employed for pediatric intensive care medicine

- yes, for the patients, on a consultant basis

- yes, also for family members, a ward psychologist employed by the PICU

- yes, also for family members, on a consultant basis

- no

- other:

E4) Is there an aftercare program in the PICU where you work at?

- no

- yes, a handover checklist

- yes, through visitation after transfer from PICU

- yes, telephone / electronic (Email) follow-up / consultation

- yes, a follow-up outpatient clinic (with participation of staff from the PICU)

- yes, home visits (with participation of staff from the PICU)

- other:

E5) Which of the following (maximum 3) factors do you perceive as the most important obstacles to the implementation and / or daily operation of aftercare programs?

- diffusion of responsibility / unclear allocation of tasks between the outpatient and the inpatient sector

- lack of personnel

- lack of time

- lack of premises

- lack of financial support

- lack of awareness of the treatment team for the need for aftercare following intensive care treatment

- lack of necessity on the part of parents / children

- I do not know

- other:
